# Numerical Evaluation on Residual Thermal Stress-Induced Delamination at PDMS–Metal Interface of Neural Prostheses

**DOI:** 10.3390/mi12060669

**Published:** 2021-06-08

**Authors:** Yuyang Mao, Ivan Pechenizkiy, Thomas Stieglitz, Theodor Doll

**Affiliations:** 1 BioMaterial Engineering, Department of Otorhinolaryngology, Hannover Medical School, Carl Neuberg-Str. 1, 30625 Hannover, Germany; maoyy200561@163.com (Y.M.); pechenizkiy.ivan@mh-hannover.de (I.P.); 2 Laboratory for Biomedical Microtechnology, Department of Microsystems Engineering-IMTEK & BrainLinks-BrainTools Center, University of Freiburg, 79110 Freiburg, Germany; stieglitz@imtek.de; 3 Fraunhofer Institute for Toxicology and Experimental Medicine ITEM, Nikolai-Fuchs-Str. 1, 30625 Hannover, Germany

**Keywords:** residual stresses, delamination, adhesion, polydimethylsiloxane-PDMS, shrinkage, neural electrodes, neural implant

## Abstract

The most common failure mode of implantable neural implants has been delamination of layers in compound structures and encapsulations in a wet body environment. Current knowledge of failure mechanisms of adhesion and its standardized test procedures are lacking and must be established. This study demonstrated a combined experimental and numerical method to investigate the residual stresses from one of the most common encapsulation materials, silicone rubber (polydimethylsiloxane-PDMS) during the coating process at elevated temperatures. Measured shrinkage of test specimen correlates well to a modified shrinkage model using thermal-mechanical finite element method (FEM) simulation. All simulated interfacial stresses show stress concentration at the PDMS coating front depending on curing temperature and coating thickness, while Griffith’s condition estimated the delamination of the coating front. This study emphasizes the understanding of the interfacial delamination giving the possibility to predict failure mode of neural interface.

## 1. Introduction

Neural prostheses or Active Implantable Medical Devices (AIMD) replace or restore disabled body functions with successful clinical applications like the cochlear implant (CI), electrocorticography (ECoG) electrodes, or deep brain stimulation. Their implantable electrodes serve as a neural interface by stimulating or receiving electrical impulses with the human nervous system. For example, platinum–iridium alloys in CIs’ flexible electrodes are typically selected as the conductor in a soft, stretchable coating, for which soft silicone rubber (polydimethylsiloxane-PDMS) is the most common choice. After implantation, the electrode’s compound material structure works in a complex moist physiological environment, in which delamination has been considered the primary failure mode [1].

The complex mechanisms of delamination have not been thoroughly studied and could be contributed by various factors. As for its biological and electrochemical aspects, the change of the electrode contact zone’s morphology by cell adhesion [2] and surface corrosion [3] have already been experimentally confirmed. The physical adhesion is the only known interaction to ensure the stable PDMS–metal interface against delamination since it is impossible to build a long-term stable chemical compound between a polymer and alloys. Nonetheless, this adhesion is known to be weak and is often absent for several years after immersion in body fluid. For the devices’ integrity and durability, metal conductor must adhere to the surrounding coating layer. When delamination occurs (i.e., adhesion fails), layers with a thickness of less than one micrometer fail instantly [4]. Metal foils and wires are mechanically stable enough to maintain function but delamination might eventually lead to short-circuits in between lines and corrosion-induced electrical failure [5]. Advances have been made to strengthen the poor interfacial adhesion in the PDMS–metal interfacial joint. For instance, by applying an additional adhesion promoter between layers [6], plasma-treated polymer surfaces [7] or interlocking via the perforation holes [8].

**Figure 1:**
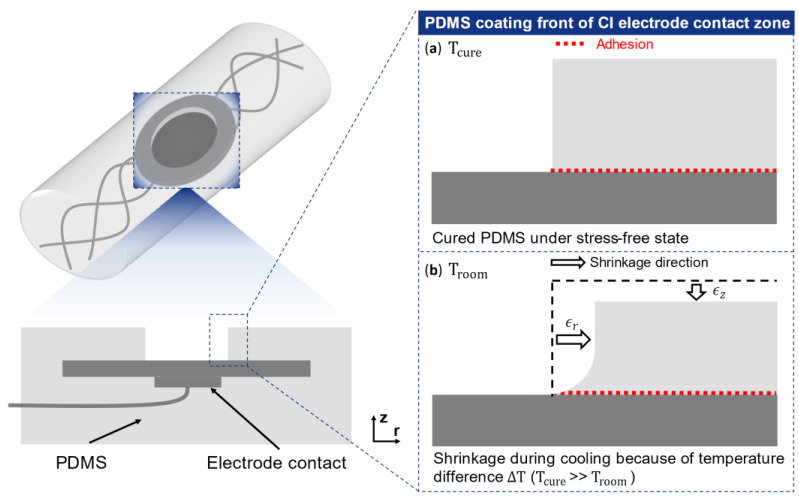
Schematic view of the PDMS coating’s shrinkage at the electrode’s contact zone of cochlear implant (CI): (**a**) under high curing temperature (e.g., 120 °C), the liquid PDMS crosslinks to an isotropic elastic solid (curing) with a coefficient of thermal expansion α. (**b**) After cooling from high-curing temperature $T_{cure}$ down to the room temperature $T_{room}$, the coating’s linear shrinkage strain in axial ($\epsilon_{z}$) or radial $\left(\epsilon_{r}\right)$ direction, where $\;\epsilon_{z/r}=\Delta T\cdot \alpha$ is induced by the temperature difference ΔT ($\Delta T=T_{cure}-T_{room})$.

However, none of these techniques can ensure the desired long-term stable bonding and the delamination could still be initiated by the inherent thermoelastic residual stresses “frozen” in the PDMS coating. Residual stress is one of the many experimental parameters that describes delamination mechanisms [1]. Besides, under implantation conditions, interfacial stresses lead to accelerated fatigue between the highly extensible elastomers and the stiff metal conductors [9], for example in the electrodes. Thus, it is crucial to investigate the origin and the role of residual stress in interfacial delamination. Parts of these residual stresses relate to PDMS’s shrinkage and its processing history. The current standard process in the manufacture of neural implants is still thermal casting [10], where PDMS shrinks due to thermal expansion after cooling from high curing temperature to room temperature [11], which is illustrated schematically in Figure 1. The different coefficients of thermal expansion (CTE) of PDMS (we use Sylgard 184 in this study) and metal (e.g., copper) result in a mismatched strain in a difference in environmental temperature, thus residual stress in the interface. Besides, the mechanical properties of PDMS show a dependency on the curing temperature [12,13], so that a simple linear model cannot accurately predict PDMS’s shrinkage.

This study demonstrates a method to evaluate the residual stresses generated during PDMS processing experimentally and numerically to assess the risk of delamination at the interface. A casting system was designed to mass-produce the test specimens representing the implantable electrode’s PDMS–metal interface. A polished, rigid copper disk was applied as the conductor contact in the specimen’s fabrication, avoiding the influence of surface roughness on adhesion. Copper was chosen due to cost aspects well knowing that the material will never be used in implants due to the material’s toxicity. Shrinkage results were measured from these specimens compared to a modified shrinkage model from other authors that correlate to that of thermal-mechanical finite element analysis. Finally, the electrode contact zone’s stability against delamination is estimated by Griffith’s condition.

## 2. Materials and Methods

### 2.1. Test Specimen Fabrication

The fabricated test specimen consists of two materials: a copper disk equivalent to the electrode’s metal conductor and the PDMS as the coating material prepared according to the product data sheet [14]. Component A (pre-polymer) and B (curing agent) of the two-part PDMS Sylgard 184 (Dow Chemical) were mixed by a speed mixer (Hauschild GmbH) in a ratio of 10:1. The mixture was further degassed under 100 mbar for at least 10 min afterwards and prepared for the casting procedure. The concentric copper disk is 1 mm thick with an inner diameter of 9 mm and an outer diameter of 70 mm.

**Figure 2:**
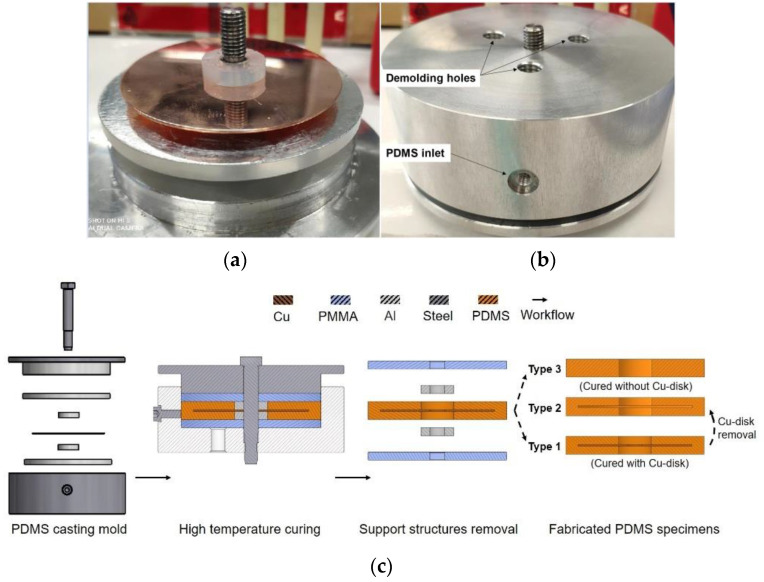
Designed casting mold for the fabrication of test specimens. (**a**) De-assembled mold (**b**) Bottom side of the mold with a PDMS inlet and three demolding holes (**c**) Fabrication of three types of PDMS specimens. Type 1: PDMS coating cured with copper substrate represents the PDMS encapsulated electrode metal contact; Type 2: Carefully cutting the coating of Type 1 specimen from one side and removing the copper substrate (Cu-disk); Type 3: PDMS specimen cured without the copper substrate.

The actual casting process of the CI-electrode was replicated in a macro size (about 100 times larger than the real CI electrode contact zone), where test specimens were fabricated by casting the PDMS in a self-designed mold. The key advantages of the casting process are its low cost and the possibility for mass production while obtaining a clear, defined PDMS front at the PDMS–metal interface. One additional advantage of implementing the symmetric macro-sized specimens is to avoid the high fabrication cost of microsized specimens, yet allowing efficient for modelling and simulation procedure by the later finite element analysis. The test specimens were designed and fabricated based on a stepwise workflow (see Figure 2c). The prepared PDMS mixture was cast in the mold and transferred to a thermal chamber (Memmert, Typ U-30, Apeldoorn, The Netherlands) at various curing temperatures (see FEM simulated curing protocol in Table 1). The proposed curing protocol was based on a thermal simulation result considering the compound material structure in the casting mold. After curing, the specimens were de-molded and categorized into three different types for later shrinkage measurement.

### 2.2. Shrinkage Measurement Experiment

**Figure 3:**
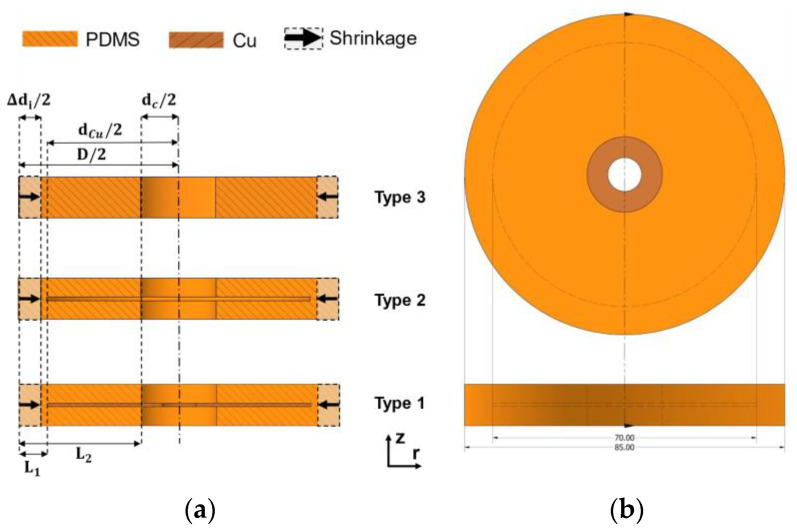
Shrinkage measurement setup. (**a**) The lateral shrinkage $\Delta d_{i}$ (i = 1,2,3) in the radial direction of three types of specimens where an original diameter of D=85 mm was measured. Meanwhile, the diameter of the electrode contact zone and the copper disk is $d_{c}$ =28 mm and $d_{Cu}$ = 70 mm, respectively. (**b**) The measurement was designed to be performed in 2D cross-section from the specimen’s 3D view.

The use of 2D lateral measurements is a well-established approach in measuring PDMS’s shrinkage as a coating material [11]. To examine the possible mismatch strain in the PDMS–metal interface, the lateral shrinkage of PDMS coating for each type was measured multiple times (n = 10) by a precise digital caliper (Scala Messzeuge GmbH, Dettingen, Germany) with two decimal digits accuracy. Figure 3 shows a schematic measurement of three different types of specimens. The inner diameter *D* of the mold is 85 mm without shrinkage, while the measured outer diameters of shrunk types 1 to 3 specimens are $d_{i}$ (*i* = 1, 2, 3). Hence the measured lateral thermal deformation $\Delta d_{i}$ is:

(1) $$\Delta d_{i}=\left|d_{i}-D\right|$$

### 2.3. Modified Thermal Shrinkage Model for Correlation

The thermal deformation model of PDMS Sylgard 184 was reported by Mueller et al. [12]. It was modified in this study to verify the shrinkage measurement results. The thermal shrinkage of the isotropic PDMS Sylgard 184 is considered as a small deformation so that the linear elastic mechanics model applies. Hence its strain and stress can be described by Hook’s law:

(2) $$\varepsilon_{i}=D_{i,j}\sigma_{j}$$

where the linear elasticity tensor $D_{i,j}$ can be expressed with Young’s modulus *E* and Poisson’s ratio ν:

(3) $$D_{i,j}=\frac{1}{E}\cdot \left[\begin{array}{cccccc} 1 & -v & -v & 0 & 0 & 0\\ -v & 1 & -v & 0 & 0 & 0\\ -v & -v & 1 & 0 & 0 & 0\\ 0 & 0 & 0 & 2+2v & 0 & 0\\ 0 & 0 & 0 & 0 & 2+2v & 0\\ 0 & 0 & 0 & 0 & 0 & 2+2v \end{array}\right]$$

For small deformation, the shear components in the elasticity tensor are ignored. Moreover, Sylgard 184’s polymerization shrinkage was neglected [15] so that its deformation is dominated by thermal expansion:

(4) $$\vec{\varepsilon }=\frac{1}{E}\left[\begin{array}{ccc} 1 & -v & -v\\ -v & 1 & -v\\ -v & -v & 1 \end{array}\right]\vec{\sigma }+\left[\begin{array}{c} 1\\ 1\\ 1 \end{array}\right]\alpha \Delta T$$

where α is PDMS’s linear coefficient of thermal expansion, and Δ*T* is the temperature difference between the curing and the room temperatures. For the cylinder specimens shown in Figure 3, the bottom and top surfaces are assumed to be not deformable under pressure so that fixed ($\epsilon_{z}=0$). In contrast, the cylinder sidewall is free for radial shrinkage ($\sigma_{r}=0$) in the cavities of casting mold. Hence the tangential stress $\sigma_{z}$ becomes:

(5) $$\sigma_{z}=-\alpha \Delta TE$$

Substituting Equation (5) into the strain Formulation (4), so the strain in radial direction becomes:

(6) $$\varepsilon_{r}=(v+1)\alpha \Delta T$$

Hence, for an asymmetric deformable radial length $L_{i}$ of the deformable part in the coating, its linear lateral shrinkage deformation $\Delta l_{i}$:

(7) $$\Delta l_{i}=\varepsilon_{r}\cdot L_{i}$$

Two different $L_{i}\;$ were used later for the validation (see Figure 3). The type 1 specimen has a lateral deformable length $L_{1}$ of 7.5 mm calculated by inner diameter *D* of the mold and the outer diameter $d_{Cu}$ of 70 mm of the copper disk. Similarly, the free deformable length $L_{2}$ of 28.5 mm for type 2 and 3 specimens was computed by the contact zone diameter $d_{c}$ of 28 mm:

(8) $$\begin{array}{l} L_{1}=\frac{1}{2}\cdot \left(D-d_{Cu}\right)\\ L_{2}=\frac{1}{2}\cdot \left(D-d_{c}\right) \end{array}$$

### 2.4. Finite Element Method (FEM) Simulations

The commercial finite element package ANSYS workbench 20.2 was used to perform the thermomechanical analysis. The relevant governing equations are similar to that of Section 2.3 in matrix form. This section describes the simulation setup and processes, and the simulation procedure consists of three general steps:

- Creation of the finite element model and perform the thermal simulation.
- Definition of the boundary and contact constraints and thermal simulation results as inputs for mechanical simulation.
- Post-processing and evaluation of the simulation results.

**Figure 4:**
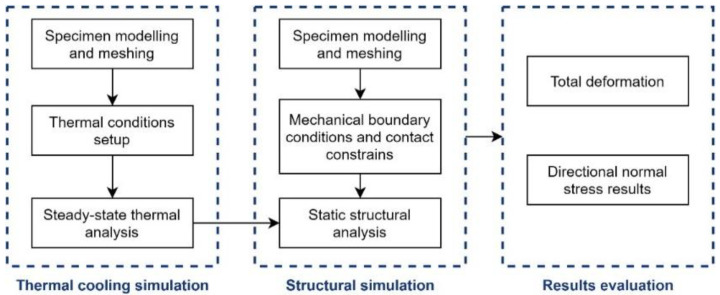
Flowchart of the finite element method (FEM) simulation procedure.

**Figure 5:**
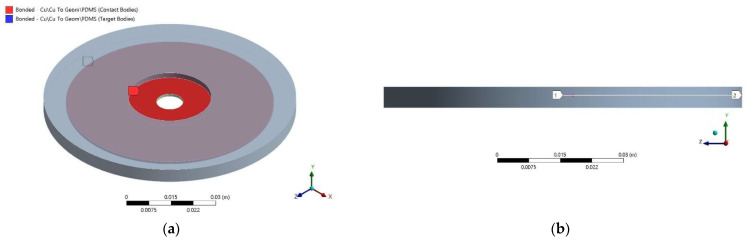
Finite element simulation setup: (**a**) contact constraints for the test specimen (**b**) the simulated normal stresses in z-direction along a construction path are evaluated in post-processing.

A summarized schematic workflow of the detailed simulation setup is shown in Figure 4. The material parameters and a temperature-dependent material model of PDMS Sylgard 184 were defined, including its mechanical properties like the coefficient of thermal expansion (CTE) [12] Young’s modulus [13] (see Table 2). The specimen was modelled and meshed with a mesh size of 3 mm to ensure an accurate and efficient solution. Assuming that each specimen was sufficiently cured, the thermal simulation covered the PDMS coating’s cooling process from various curing temperatures (e.g., 120 °C) down to the room temperature (22 °C). For the structural simulation, a “fixed” boundary condition at the inner ring of the copper substrate and “bonded” contact constraints were imposed into the mechanical model (see Figure 5a), assuming an intact interfacial adhesion without delamination of the coating. 

The thermal–mechanical coupling was achieved by adopting the simulated thermal history as loading for the structural simulation. The coupled simulation was solved with the default APDL solver, while “total deformation” and “normal stress” were chosen as the results to evaluate in the post-processor. The normal stress distribution was solved along a defined “path” near the interfacial zone located 0.005 mm above the copper substrate geometry (see Figure 5b) representing the residual thermal stress at the specimen’s interfacial zone.

## 3. Results and Discussions

### 3.1. PDMS Lateral Shrinkage

This study directly observed PDMS coating’s shrinkage and measured the strain mismatch at the electrode contact zone between different test specimen types based on the lateral measurement method. Various designs of the test specimens for the study of PDMS’s shrinkage were considered, including the most applied dumbbell shape [13,16]. However, the circular substrate geometry used in this study has three advantages. First, circular geometry is the easiest to calculate analytically. Second, the angular measurement provides the same detail concerning the distance to the center, and third, the corners of non-circular specimens cause an inhomogeneous stress distribution.

**Figure 6:**
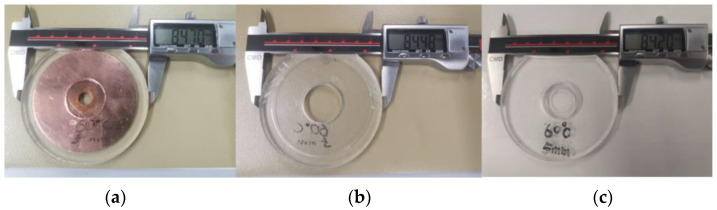
Example results of the experimental shrinkage measurement by a digital caliper of a specimen (coating thickness $h_{C}$ = 2.5 mm) cured at 60 °C. (**a**) Measured Type-1 diameter $d_{1}$ =84.70 mm. (**b**) Measured Type-2 diameter $d_{2}$ =84.48 mm. (**c**) Measured Type-3 diameter $d_{3}$ = 84.20 mm.

**Figure 7:**
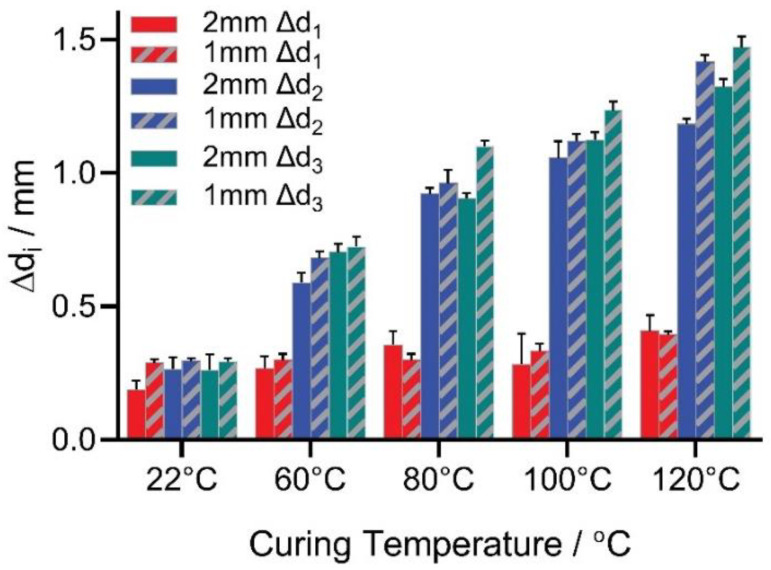
Shrinkage measurement results of three types of test specimens. Lateral thermal deformation $\Delta d_{i}$ at coating thicknesses of 1 mm and 2 mm for Sylgard 184 at various curing temperatures (n = 10 measurements per specimen).

Figure 6 shows an example measurement result of a test specimen cured at 60 °C. In contrast, Figure 7 summarizes the experimentally measured lateral shrinkage of all three specimen types of different coating thicknesses (1 mm and 2 mm) and curing temperatures. The results demonstrate that curing temperatures, as predicted, correlate positively to the PDMS coating’s lateral thermal shrinkage. 

However, the type 1 shrinkage $\Delta d_{1}$ in red is not as significant comparing to the other types, $\Delta d_{2}$ and $\Delta d_{3}$. In this case, the stiff copper disk inside the type 1 specimen limited the free deformable bulk volume. The second type shrinkage $\Delta d_{2}$ is slightly smaller than the third type $\Delta d_{3}$. Although the type 2 specimen’s PDMS coating was not under restriction after removing the copper disk, the cured cavity was left behind, resulting in stress concentration near the irregular cavity edges. Moreover, compared to the 2-mm thick specimen, the higher values of the 1-mm-thick specimen at all curing temperatures indicate that the coating’s thickness could play a role in the shrinkage and was investigated and discussed in later sections.

The measurement was repeated ten times (n = 10) for each group, in which the narrow error ranges indicate a reliable experimental measurement (See Table 3 for detailed measurement data). While curing at room temperature was reported to deliver a precious PDMS fabrication without shrinkage [17], our measured shrinkage results at room temperature were erroneous. Unlike the difference between measurement results at higher curing temperatures, shrinkages of about 0.25 mm were measured for all three types of specimens at 22 °C. This phenomenon indicates an inherent tolerance in the mold parts because of workpiece processing, which will be calibrated and validated in the later section.

### 3.2. Validation of the Shrinkage Measurement Results

**Figure 8:**
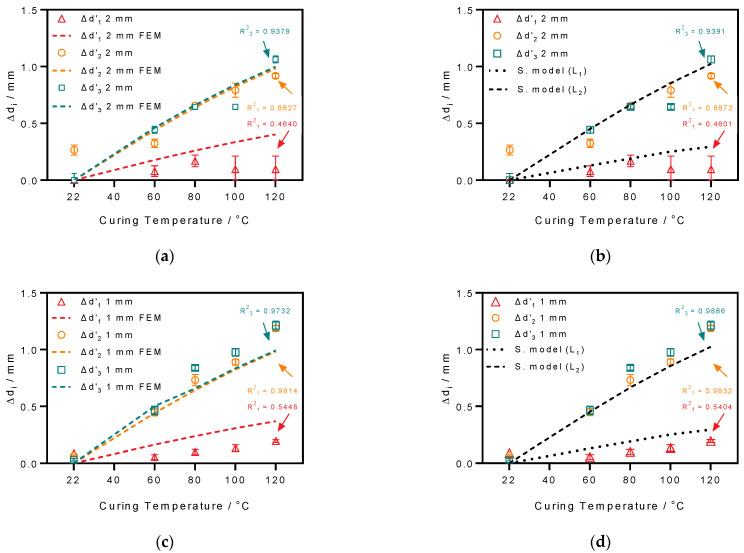
Validation of the calibrated shrinkage measurement results for 2-mm and 1-mm-thick coatings by fitting that of the FEM thermomechanical simulation and the modified shrinkage model. The results of the 2-mm-thick specimen coating validated by (**a**) FEM simulation and (**b**) the shrinkage model. The results of the 1-mm-thick specimen coating validated by (**c**) the FEM simulation and (**d**) the shrinkage model. (All correlation coefficients $R^{2}$ are calculated with GraphPad Prisim 7.0).

PDMS Sylgard 184′s coating should not undergo any thermal shrinkage when cured at room temperature [15] according to Equation (4). Therefore, the experimentally measured shrinkage results (see Figure 7) were calibrated by:

(9) $$\Delta d_{i}^{\prime }\left(T_{i}\right)=\Delta d_{i}\left(T_{i}\right)-\Delta d_{i}\left(22\;{}^{\circ}C\right)$$

where $\Delta d_{i}\left(T_{i}\right)$ is the measured shrinkage at curing temperature $T_{i}$ subtracting the shrinkage at room temperature (22 °C) so that the calibrated result is $\Delta d_{i}^{\prime }\left(T_{i}\right)$. For instance, the calibrated shrinkage at 60 °C is: $\Delta d_{i}^{\prime }\;\left(60\;{}^{\circ}C\right)=\Delta d_{i}\;\left(60\;{}^{\circ}C\right)-\Delta d_{i}\;\left(22\;{}^{\circ}C\right)$. The correlation method was applied to validate the measurement results and predict the shrinkage behavior. Figure 8 illustrates the calibrated shrinkage results $\Delta d_{i}^{\prime }\left(T_{i}\right)$ of different coating thicknesses at various curing temperatures, compared with the modified correlation model proposed by Mueller et al. (see Section 2.3) and the conducted finite element simulation.

Generally, the performed thermal-mechanical FEM analysis considering temperature-dependent material parameters is partly reliable because it correlates with the modified thermal shrinkage model (the square of the correlation $R^{2}$, see Figure 8). The coating’s shrinkage of type 2 and 3 results of the 1-mm specimen are more precisely correlated by the model and simulation 2-mm specimen, while poor correlation agreement can be observed in all type 1 results. The differences between simulation and measurements in type 1 results might come from device handling during measurements. As for the 2-mm-thick specimen, the results of type 2 and 3 at high curing temperatures, like 120 °C, are biased against its FEM results. The heat transfer within specimens with thick coating may cause uneven curing, thus affecting the measured shrinkage results.

### 3.3. Simulated In-Plane Residual Stress Distribution in PDMS-Metal Interface

**Figure 9:**
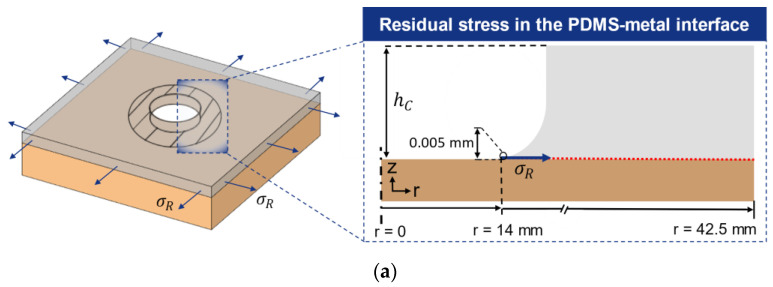

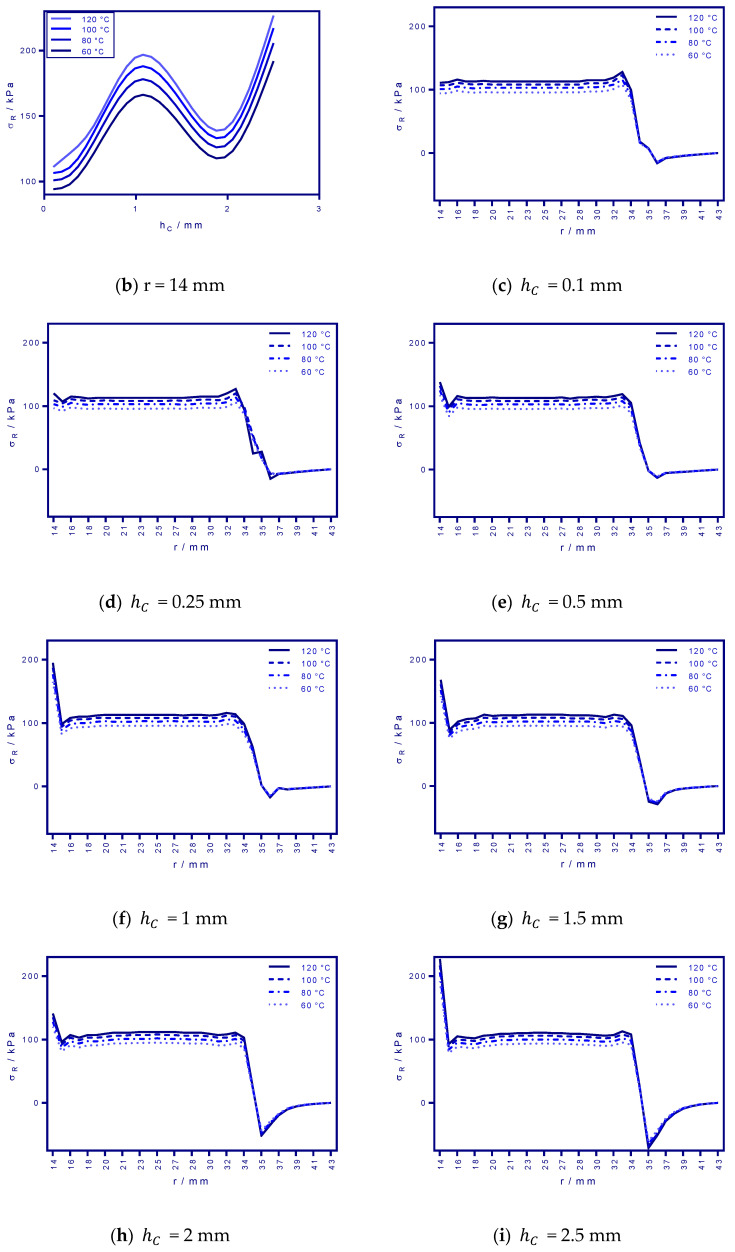
Residual in-plane stress in the PDMS–metal interface at various coating thicknesses and curing temperatures. (**a**) Schematic view of the shrunk PDMS coating front and (**b**) residual stress distribution $\sigma_{R}$ from the circular free edge in PDMS coating (r = 14 mm) with thicknesses $h_{C}$ of (**c**) 0.1 mm (**d**) 0.25 mm (**e**) 0.5 mm (**f**) 1 mm (**g**) 1.5 mm (**h**) 2 mm (**i**) 2.5 mm.

Figure 9a illustrates the shrinkage of the coating front under an in-plane residual thermal stress $\sigma_{C}$ assuming an intact physical adhesion force at the PDMS–metal interface. The residual stresses are evaluated in the post-processor of ANSYS with various coating thicknesses $h_{C}$ and positions r in radial direction along the interface.

Figure 9b shows that residual stress increases as the coating thickness of the test specimen rise, while it is somewhat surprising that extraordinarily high stress occurs at the 1-mm-thick coating. This finding is consistent with the poor correlation of the 1 mm specimen results, in which the simulated shrinkage is more significant than the measured ones. Moreover, some authors claimed that PDMS parts might not be homogeneously cured because of the heat transfer [15] issues and the size of the PDMS part [18]. Considering the composite material structure of the molding system, the shrinkage of the unevenly cured 2 mm specimen could be hence diminished compared to the 1-mm-thick specimen. Nonetheless, the coating’s thickness correlates positively with its stress level. High value occurs at the beginning (r = 14 mm) of the stress distributions of various PDMS coating thickness (see Figure 9c–i), indicating the risk of delamination initiation at the coating front.

### 3.4. Estimation of Delamination Driven by Residual Stress

In order to predict and estimate the advance of delamination at the PDMS–metal interface, the elastic deformation energy $G_{\epsilon }$ of shrunk PDMS coating was computed from residual stress distribution $\sigma_{R}$. Along the radial direction at various curing temperatures, according to Griffith’s condition [19]:

(10) $$G_{\varepsilon }=\frac{1-{v_{c}}^{2}}{2E_{c}}\sigma_{R}^{2}h_{c}$$

where $h_{c}$ is the film thickness of the coating with elastic modulus $E_{c}$ and Poisson’s ratio $\nu_{c}$. By comparing the elastic deformation energy of shrunk PDMS coating computed by Equation (10) and the physical adhesion energy $\Gamma_{ad}$ of PDMS to metal (1 J/m^2^ [20,21]), the delamination of the PDMS-copper interface can be predicted. The delamination at the coating front should happen if the deformation energy is greater than the adhesion energy at the interface [6]:

(11) $$\begin{array}{c} G_{\varepsilon }-\Gamma_{ad}>0\;\;\;\;\;\;\;\;\mathrm{adhesion}\\ G_{\varepsilon }-\Gamma_{ad}\leq 0\;\;\;\;\;\mathrm{delamination} \end{array}$$

**Figure 10:**
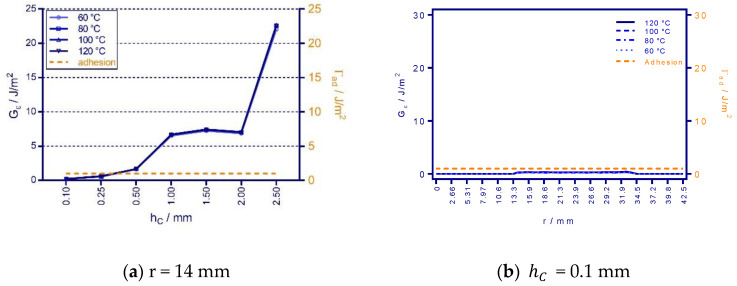

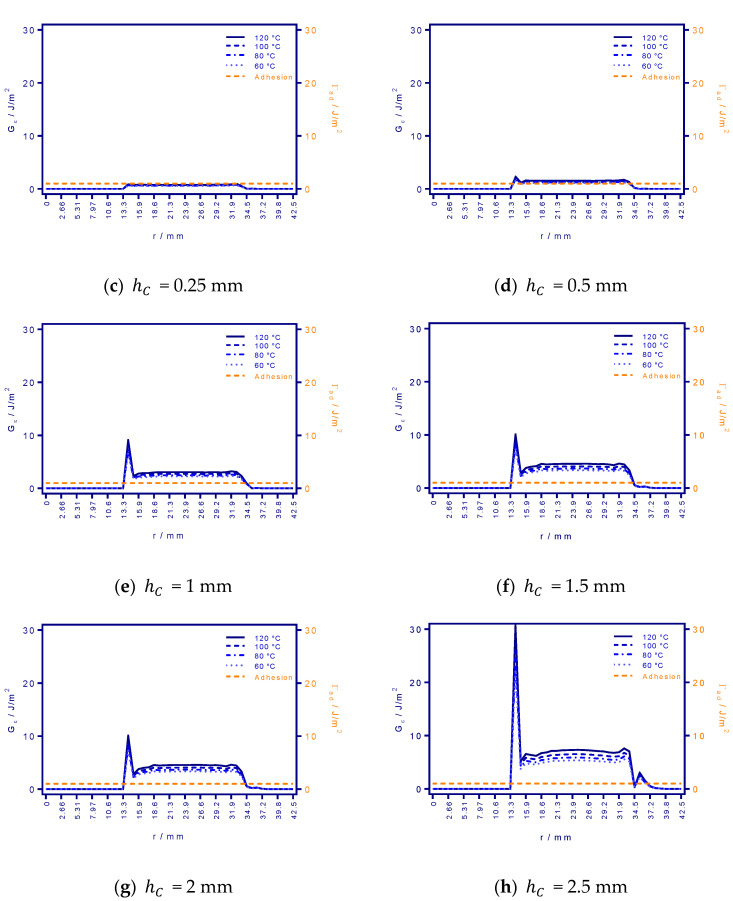
Comparison of elastic deformation energy to the physical adhesion energy of the PDMS–copper interface after shrinkage. (**a**) Deformation energies at the circular free edge of PDMS coating (r = 14 mm) and the evaluated results of PDMS coating with thicknesses $h_{C}$ of (**b**) 0.1 mm (**c**) 0.25 mm (**d**) 0.5 mm (**e**) 1 mm (**f**) 1.5 mm (**g**) 2 mm (**h**) 2.5 mm. Values larger than the adhesion force (dashed orange line) lead to delamiantion.

Figure 10a shows an overall view of deformation energy $G_{\epsilon }$ against the adhesion energy $\Gamma_{ad}$ of PDMS–metal interface at the coating front (r = 14 mm) and agrees to the stress distribution shows high deformation energy from the 1-mm-thick coating. It demonstrates a stable interfacial bonding for thin coating when $h_{C}$ is smaller than 0.25 mm. However, for thicker coating, the delamination at the coating front will be initiated against the weak adhesion force according to the proposed criterium.

## 4. Conclusions

The residual thermal stress is one of the many factors that has been discussed and believed to initiate the delamination of the neural interface. However, not many studies investigated residual stress quantitatively and its influence on interfacial stability. This study starts by measuring the thermal shrinkage generated during the electrode’s fabrication process based on equivalent test specimens under the assumption that the specimens’ size does not alter the interfacial adhesion mechanisms. The different shrinkage between designed specimen types demonstrates a thermal mismatch at the PDMS–metal interface, which correlates well with the modified thermal shrinkage model results and the developed finite element simulation. The simulation result indicates a concentration of residual stresses at the PDMS coating front depending on the coating’s thickness and the curing temperature. Griffth’s condition estimated delamination initiation at the coating front by comparing the elastic deformation energy of the shrunken coating and the adhesion energy of the interface. The high curing temperature and thick PDMS coating-induced stresses will result in delamination at the PDMS–metal interface.

The combined experimental and numerical simulation was applied to understand the formation of residual thermal stress in the PDMS–metal interface. This study provides insight for optimizing the PDMS fabrication process, which further leads to a deeper understanding of the interfacial delamination and contributes to predicting the device failure of neural interface.

## Figures and Tables

**Table 1:** Curing protocols of Sylgard 184 by the FEM thermal simulation compared to different sources.

Curing Temperature (°C)	25	40	60	80	100	120
Curing protocol by FEM simulation (min)	833	510	180	150	120	105
Sylgard 184 data sheet (min)	2880	-	-	-	35	25
Mueller et al. [12]	-	360	-	-	-	18

**Table 2:** Material properties for PDMS Sylgard 184 and copper. * Constant at all chosen temperatures for PDMS Sylgard 184.

Property	Copper	PDMS Sylgard 184				
		22 °C	60 °C	80 °C	100 °C	120 °C
$\mathrm{Density}\;(\mathrm{kg}\cdot m^{-3}$)	8942	982 *				
$\mathrm{Specific}\;\mathrm{heat}\;\mathrm{capacity}\;(J\cdot {\mathrm{kg}}^{-1}\cdot K^{-1}$)	385	1100 *				
$\mathrm{Thermal}\;\mathrm{conductivity}\;(W\cdot m^{-1}\cdot K^{-1}$)	401	0.27 *				
Poisson’s ratio	0.345	0.495 *				
$\mathrm{Coefficient}\;\mathrm{of}\;\mathrm{thermal}\;\mathrm{expansion}\;\cdot 10^{-6}\left(K^{-1}\right)$	16.74	337.50	312.70	298.53	284.36	270.19
Elastic modulus (MPa)	$1.26\cdot 10^{5}$	1.320	1.577	1.770	1.962	2.155

**Table 3:** Shrinkage measurement data of three types of test specimens (n = 10).

Shrinkage Types	22 °C (mm)	60 °C (mm)	80 °C (mm)	100 °C (mm)	120 °C (mm)
$2\;\mathrm{mm}\;\Delta d_{1}$	0.187 ± 0.034	0.267 ± 0.047	0.356 ± 0.051	0.284 ± 0.115	0.408 ± 0.059
$1\;\mathrm{mm}\;\Delta d_{1}$	0.289 ± 0.012	0.299 ± 0.023	0.299 ± 0.023	0.333 ± 0.028	0.394 ± 0.013
$2\;\mathrm{mm}\;\Delta d_{2}$	0.265 ± 0.045	0.589 ± 0.036	0.922 ± 0.022	1.057 ± 0.063	1.183 ± 0.021
$1\;\mathrm{mm}\;\Delta d_{2}$	0.296 ± 0.008	0.682 ± 0.026	0.962 ± 0.049	1.120 ± 0.027	1.417 ± 0.027
$2\;\mathrm{mm}\;\Delta d_{3}$	0.261 ± 0.058	0.704 ± 0.030	0.905 ± 0.020	1.123 ± 0.030	1.323 ± 0.030
$1\;\mathrm{mm}\;\Delta d_{3}$	0.293 ± 0.012	0.724 ± 0.038	1.099 ± 0.021	1.235 ± 0.034	1.472 ± 0.040

(Values are given as mean ± SD.).

## Abbreviations

AMID	Active Implantable Medical Devices
ECoG	electrocorticography
PDMS	polydimethylsiloxane
CI	cochlear implant
CTE	coefficients of thermal expansion
FEM	finite element method
APDL	ANSYS Parametric Design Language
